# An Inquiry into the Contagious Property of Hooping Cough

**Published:** 1810-03

**Authors:** J. Adams


### Medical and Physical Journal.

VOL. XXIII.] MARCH, 1810. [NO. 133.

*Printed for R. PHILLIPS, by W. Thorne, Red Lion Court, Fleet Street, London.*

### AN INQUIRY INTO THE CONTAGIOUS PROPERTY OF HOOPING COUGH. By J. ADAMS, M. D.

UNDER whatever artificial distinctions we attempt to confine Nature, we invariably encounter difficulties. They seem to teach, or rather to remind us, that though order may every where be perceived in the physical constitution of things, yet that it is in vain for us to attempt comprehending the whole design. In our division of the different kingdoms of nature, the different classes of animals, and the different varieties in each class, after all our labours, we arrive at a step which, instead of attempting to surmount, we are obliged to leave as a memorial of our own weakness.

If such is the case in the ordinary appearances of nature, how much greater must our difficulties be in attempting to reduce to order those transient appearances which disease assumes? Even here, however, we discover some general uniformity, which flatters our vanity by inducing us to suppose that we have detected a law; and though we often find ourselves mistaken, yet the detection of that very error brings us nearer to the truth.

I have been led to these reflections by the attempts which have for some time engaged me to ascertain the various kinds and degrees of contagion. For this purpose, the first object was to fix on a certain definition. Such diseases as are generally admitted to be contagious, agree in the following points. 1st. That as far as our knowledge extends, they can only be excited by matter secreted during the disease. 2dly, That every person under the influence of them is contagious either in the part affected, or by the effluvia from some part of his body. 3dly, That he renders those who are susceptible of the same disease, contagious also. 4thly, That the effect is confined to no climate, season, or temperature. 5thly, That if the disease produces a constitutional effect, the person is for ever after insensible to that effect. The contagions in which all these laws are found with great regularity, are small-pox, measles, chicken-pox, and scarlatina.

There are others in which the contagious property is more doubtful; the plague and yellow fever, whether contagious or not, are only found to exist under certain temperatures, and at certain seasons, and the constitution remains still susceptible of each. Dysentery seldom spreads but at certain seasons, among men particularly situated, and leaves the subject as susceptible as before. What is vulgarly called typhus fever, though confined to no seasons, yet preserves the same law in leaving the subject for ever susceptible of the disease. All these may likewise be excited without the presence of matter secreted by another under the same disease.

These distinctions are so permanent, that if they are not sufficient to prove plague, yellow fever, dysentery, and typhus are not contagious, they at least show that their contagious properties are different from those of small-pox, measles, and scarlatina; and this difference is so permanent as to be universally admitted. But there is one disease which attacks a person only once during life, which seems confined to no temperature, season, or climate, and which when it occurs spreads generally among those who remain susceptible of it. In all these respects, hooping cough seems to have the property of a true contagion, yet it is often difficult to trace it from any certain source; and the instances in which, at one time, a diseased subject seems to prove contagious, at others perfectly innocuous, are much more numerous than in the small-pox, measles, or scarlatina.

In the latter end of the year 1806, the second edition of "Morbid Poisons" was published, and contained the following remark, or rather inquiry. "The hooping cough is attended with difficulties in the mode in which it subsides and returns, that I pretend not to account for. In many respects it seems to partake of the endemics, aided by a peculiar constitution of the air; and the advantages derived from a change of atmosphere would seem to strengthen this opinion: but I have not satisfied myself of the manner in which this disease arises and seems communicated."

The last number of Dr. Clutterbuck's Review, published at the beginning of 1808, gives an

"*Account*

"*Account of the spontaneous Appearance of Hooping-Cough.* By Dr. A. C. Willey, of Block Island (N. America).

"The *Tussis Convulsiva*, or Hooping-Cough, occurred here in April 1805, and did not become wholly extinct till some time in Autumn. What rendered it particularly worthy of attention, was its being indigenous. It made its appearance over the greater part of the island at the same time, and was untraceable to any apparent source. The insulated situation of this place is extremely favourable to observations, and the detection of facts of this nature, without the danger of deception; and has afforded, in the present instance, a fair demonstration that the hooping-cough can originate without contagion. Indeed, I am inclined to believe that the rise and progress of this epidemic disease do not depend so much upon contagion as is generally imagined. The universal belief that the system, during the operation of *pertussis*, generates a specific virus capable of communicating the disease, seems to have prevented the mind from looking any farther for a principle adequate to its production. But because the living system possesses the power of elaborating this virus, I know of no reason why we should deny its formation in the departments of inanimate nature. The laws of physiology and inorganic matter agree in the production of soda and lime; why not then in the generation of pertussic poison? That there *is* a principle, independent of contagion, capable of inducing this complaint, I feel fully convinced, not only from its origination on this island, but from the number of cases which I have seen in those who have never been exposed to contagion. This principle undoubtedly exists in the atmosphere, which it pervades to a certain extent; but what it is, and how formed, remains a curious subject for physical research."

In the last Number of the Medical and Physical Journal is a paper from Dr. de Fretis, of Madeira, by which it appears most satisfactorily that the hooping cough was imported into that island by the British troops.

My correspondence furnishes me with instances of hooping cough in the families of two ingenious and experienced physicians. These gentlemen, whose candour is equal to their abilities, without entering deeply into the subject of contagion, furnish in the events which passed immediately under their own notice, very striking instances of the introduction of the disease in one case spreading in all directions as if by contagion, in the other sub-

 siding siding with the subject who introduced it, and without the use of any cautionary means.

Early in June, the eldest son of the first of these gentlemen came home from school, much indisposed with severe cough, accompanied with fever. One of his school-fellows had, a short time before, been removed to his friends, in consequence of his being affected with hooping cough. This led to a suspicion which was justified by the event, for the characteristic symptoms of this harrassing disorder soon manifested themselves with considerable violence. About a week afterwards, a second son, who was at the same school, returned home, affected with the same symptoms, though in a much lighter degree. His illness soon assumed the peculiar marks of the tussis convulsiva. About a week or ten days after these two youths had been at home, the two youngest children began to cough, and gradually passed through the different stages of the same disorder. A maid servant likewise, who was persuaded that she had had the hooping cough in her childhood, was nearly at the same time with the younger children seized with cough, which soon became so violent and strangulating, as to preclude all doubt respecting the nature of her complaint.

As soon as it was ascertained that the children were affected with hooping cough, the usual frequent intercourse that subsisted between this and a neighbouring family, was so regulated, as to prevent, as much as possible, any communication between the sick children and the individuals in this second family, who had not passed through the hooping cough. Unfortunately, however, it was imagined, that one of them had had this disorder, and he was not interdicted from all intercourse; he was seized with the complaint, and in the usual period it visited progressively the several children of that family, who had not previously passed through the disorder.

I think it material to mention farther, that the second son was affected with it, and recovered for more than a week before he came from school; that during this time, but before the cough became uniquivocally *hooping*, he visited, with some others of his school-fellows, Mr. ———'s family, at Layton, and played a good deal with his children. I cannot precisely state in what time, but I believe in about a fortnight after this visit, these children were affected with coughs, which gradually put on the nature and passed through the gradations of hooping cough.

From

From these events, which though related from memory, cannot but be correct, the writer does not scruple to admit his conviction that pertussis is contagious, though till then he was in doubt. In answer to my inquiries as to the manner in which the individuals might be congregated in the different houses, and also how far the disease might be endemic in the neighbourhoods of each house, he favoured me with the following account.

The school is a solitary house, situated a mile from the town, with which it has little intercourse excepting on Sunday, in the attendance on public worship. A scholar, who appears to have been first seized with the disease in the school, had previously communicated with a family under the disease, on a supposition that he had had it. On his return to school, however, he was affected with cough, which soon assumed the character of hooping cough, and from him, it was believed, that it spread itself in the seminary.

The family at Layton to which it is suspected that one of my Correspondent's sons communicated the complaint, lives in a large airy house, completely and widely detached from any other habitation. Although the occurrence of the disorder in this family was imputed to this intercourse, it must be remarked, that the owner of this house had a son at the same school.

It is not altogether certain, whether pertussis was epidemic at Walthamstow and Layton; it is rather presumed not; there might be several cases of it, but that they were not so numerous, that the disorder could correctly be said to be epidemic.

In Hackney, however, the residence of my correspondent and the other family, it was very prevalent, to such a degree, indeed, that it may be decidedly affirmed to have been epidemic.

My Correspondent has four children, to all of whom this disorder extended its influence, but with different degrees of violence and duration. A maid servant also was visited very severely by it.

Within about forty yards of his house, is another family, whose children and his younger ones are in habits of seeing and associating with each other. As soon as it was ascertained that the hooping cough was in the family, this intercourse was suspended; some watchfulness was employed, to prevent unnecessary communication between the families. The hooping cough never made its appearance in this family. Is not the exemption of these children from the influence of the disease to be referred to the adoption of these precautions.

The other physician gives the following account of the disease in his family.

"In the year 1798 this disease was very prevalent at Richmond, and all my children successively caught it; my eldest boy, then three years old, was the first that sickened; next my second boy, nearly two years old; and afterwards my little girl, at the age of seven months: the symptoms were manifest in the first of these early in the month of October, the last sickened about the middle of November; all of them had the disease severely, particularly the little girl, who was not free from it till July 1799. The early part of that year was inclement, the North East wind blew very severely, and constantly, through the whole spring.

"Towards the close of the month of April 1807, my second daughter, then eight years old, came home to London with the hooping-cough, from the school on Richmond Green, where many of the young ladies were affected with this cough; I instructed her to avoid kissing or drinking out of the same vessel that was used by my youngest son, then four years old; the child, I believe, was attentive to these instructions, and her younger brother continued free from the disease till the month of April 1809, when he brought home the disease from a writing school in the neighbourhood of the Charterhouse, to which he had been sent for an hour in the morning, for about three weeks; both these children had the disease mildly; my second daughter was perfectly recovered before the birth of a younger daughter, in the middle of July 1807 This last little girl was at home with her youngest brother during the whole of his hooping-cough; the same instructions were given him as on the former occasion to his sister, but he forgot himself more than once, and kissed his little sister. He was free from the disease in August, and she has not been affected with hooping-cough.—Is the contagion of hooping-cough less active in mild than in severe weather?"——

It must be admitted that these cases are by no means sufficient to fix the character of a disease; they are, however, inserted on account of the authenticity of the sources from which they are derived. It was my wish and intention to have collected many more facts, especially from practitioners in the country, who have the largest opportunities of tracing in insulated houses, the comparative contagion of hooping cough with small-pox, measles, or scarlatina.

latina. But on taking leave of the Journal as one of its Editors, I have been anxious to offer these hints for the consideration of Medical Correspondents, whose communications on the subject will, I doubt not, be as well received, and certainly as well attended to, as if directed to the Author of this Inquiry.

